# The natural compound fucoidan from New Zealand Undaria pinnatifida synergizes with the ERBB inhibitor lapatinib enhancing melanoma growth inhibition

**DOI:** 10.18632/oncotarget.14437

**Published:** 2017-01-02

**Authors:** Varsha Thakur, Jun Lu, Giuseppe Roscilli, Luigi Aurisicchio, Manuela Cappelletti, Emiliano Pavoni, William Lindsey White, Barbara Bedogni

**Affiliations:** ^1^ Department of Biochemistry, Case Western Reserve University School of Medicine, Cleveland, OH, USA; ^2^ School of Science, Auckland University of Technology, New Zealand; ^3^ Takis s.r.l., Rome, Italy

**Keywords:** melanoma, ERBB3, lapatinib, natural compounds, fucoidan

## Abstract

Melanoma remains one of the most aggressive and therapy-resistant cancers. Finding new treatments to improve patient outcomes is an ongoing effort. We previously demonstrated that melanoma relies on the activation of ERBB signaling, specifically of the ERBB3/ERBB2 cascade. Here we show that melanoma tumor growth is inhibited by 60% over controls when treated with lapatinib, a clinically approved inhibitor of ERBB2/EGFR. Importantly, tumor growth is further inhibited to 85% when the natural compound fucoidan from New Zealand *U. pinnatifida* is integrated into the treatment regimen. Fucoidan not only enhances tumor growth inhibition, it counteracts the morbidity associated with prolonged lapatinib treatment. Fucoidan doubles the cell killing capacity of lapatinib. These effects are associated with a further decrease in AKT and NFκB signaling, two key pathways involved in melanoma cell survival. Importantly, the enhancing cell killing effects of fucoidan can be recapitulated by inhibiting ERBB3 by either a specific shRNA or a novel, selective ERBB3 neutralizing antibody, reiterating the key roles played by this receptor in melanoma. We therefore propose the use of lapatinib or specific ERBB inhibitors, in combination with fucoidan as a new treatment of melanoma that potentiates the effects of the inhibitors while protecting from their potential side effects.

## INTRODUCTION

Although newly available therapies have improved the life expectancy of melanoma patients, they still come short at stopping the disease. Melanomas are very aggressive cancers that rely on the activity of multiple signaling pathways for their growth, survival and propagation, and adapt quickly to new treatments becoming resistant. Novel therapeutic interventions are needed to acquire better long-lasting effects.

We have recently shown that melanomas express high levels of ERBB3 and depend on its function for growth and survival [1, 2]. Specifically, we have shown that ERBB3 is highly active (phosphorylated) in up to 70% of melanomas regardless of whether they carry mutated or wild type *BRAF*, the most common oncogenic driver in melanoma [3]. ERBB3 is one of four members of the ERBB family of tyrosine kinase receptors that are often inappropriately reactivated in various cancers. All ERBB receptors function by dimerizing with members of the family to trigger growth/survival signals. ERBB3 is the only member of the ERBB family that lacks tyrosine kinase activity, and as such, requires dimerization with other tyrosine kinase receptors to trigger a signaling pathway. While ERBB3 can potentially dimerize with all members of the ERBB family, several lines of evidence show that the strongest dimer to activate survival pathways like AKT in breast cancer is the ERBB3/ERBB2 dimer [4–7]. Similarly, in melanoma, we have demonstrated that ERBB3/ERBB2 is a key survival signaling unit that acts mostly by activating AKT [2]. Additionally, ERBB3 up-regulation is also a mechanism of resistance to anti-BRAF therapies [8]; and has been shown to promote melanoma metastasis [9].

Lapatinib is a small molecule tyrosine kinase inhibitor with a similar IC50 against ERBB2 and EGFR. Lapatinib is approved by the FDA for the treatment of ERBB2 positive breast cancer patients. We have shown that lapatinib inhibits ERBB3 signaling and hampers melanoma cell survival and tumorigenesis [3]. In particular, lapatinb works better when combined with other inhibitors of key pathways in melanoma, such as γ-secretase inhibitors [3], and BRAF inhibitors [8], highlighting a cooperation among different pathways in melanoma pathogenesis. Additionally, although generally considered well tolerated, lapatinib can lead to gastrointestinal side effects such as diarrhea, nausea and vomiting that can result in some weight loss, skin rash and fatigue [10]. Hence, means to potentiate its effectiveness while improving its tolerability would be highly desirable.

Natural compounds and supplements have become part of people's daily life. Of note, over 70% of anti-cancer agents have their origin in natural sources [11]. In particular, marine derived products have sparked a great interest in recent years given the vast natural resource [11].

Fucoidan is a highly sulfated polysaccharide of brown algae with anti-inflammatory, anticoagulant, anti-tumor and anti-angiogenic activities [12–17]. Fucoidan extracted from *Undaria pinnatifida* has shown anti-cancer activity against mouse and human cancer cell lines [18–20]. Fucoidan extracted from the New Zealand *U. pinatifida* employed here, has been reported to possess better anti-cancer activity at relatively lower doses with respect to pure fucoidan [20]. The safety of fucoidan is demonstrated by a number of animal studies [21] and by the fact that fucoidan-containing food supplements or drinks have been traditionally given to cancer patients in several countries [22]. Also, recent studies have shown fucoidan can synergize with standard anti-cancer agents and/or can reduce their toxicity [23].

Here we demonstrate that fucoidan extracted from the New Zealand seaweed *U. pinnatifida* synergizes with lapatinib by doubling its cell killing capacity towards several melanoma cell lines. These effects are associated with a further reduction of AKT and NFκB activity. Specific inhibition of ERBB3 by either shRNA or a novel neutralizing antibody [24–26] in combination with fucoidan partly recapitulated these effects, reiterating the ERBB3 pathway is a major player in melanoma cell survival. Finally, we found that fucoidan, while enhancing the anti-cancer effects of lapatinib, improves the animal welfare, rescuing weight loss that often accompanies lapatinib-based therapies. Taken together, these results indicate a combination therapy involving the clinical drug lapatinib or ERBB3 inhibitors, and the natural compound fucoidan may be a novel, safer treatment option for melanoma patients characterized by increased ERBB activity.

## RESULTS

### Fucoidan extracted from New Zealand *U. pinnatifida* enhances the therapeutic effects of lapatinib

We have recently shown that up to 70% of melanomas, regardless of whether they possess mutated or wild type BRAF, show hyper-activation of ERBB3 [3] and rely on an ERBB3/ERBB2 signaling cascade to promote cell survival [2]. Indeed, lapatinib, a clinical ERBB2 and EGFR inhibitor, effectively inhibited the ERBB3/ERBB2 pathway and importantly, delayed melanoma tumor growth in both mutated and wild type BRAF cells [3]. Although effective, lapatinib only slowed down tumor growth. Hence, we sought to improve the anti-tumor activity of lapatinib while keeping its concentration within safe therapeutic doses.

**Figure 1:**
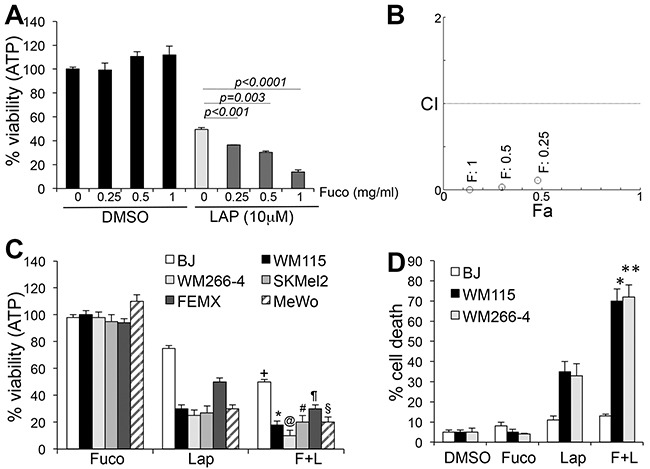
Fucoidan synergizes with lapatinib **A**. Viability of WM266-4 cells at different doses of fucoidan alone or in combination with lapatinib. **B**. Combination indexes (CI) as a function of fraction affected (Fa) (CI<1: synergism; CI=1: additive; CI>1= antagonism). **C**. Viability of BRAF^mut^ (WM115, WM266-4), RAS^mut^ (SKMel-2) and WT/WT (FEMX, MeWo) melanoma cells treated for three days with 10μM lapatinib, 1mg/ml fucoidan, alone or combined. (^+^p<0.05); (*p<0.05); (^@^p<0.001; ^#^p<0.05; ^¶,§^p<0.01. **D**. % cell death of two cell lines treated as in C (*,**p<0.0001). BJ fibroblasts were treated as the tumor cells. Results are the average of three independent experiments.

The ability of fucoidan to synergize with standard anti-cancer agents and/or reduce toxicity has recently been investigated (reviewed in [23]). We therefore tested the effects of fucoidan on WM266-4 melanoma cells and found that while fucoidan alone at different concentrations did not affect cell viability, measured as the total ATP content in cells (Cell Titer Glo Assay), it synergized with lapatinib, with the highest combinatorial effect at 1mg/ml fucoidan (Figure 1A, 1B). To determine if the synergistic inhibition of viability affected a variety of melanoma subtypes, cells with different genetic drivers were subjected to a three-day treatment with 10μM lapatinib and 1mg/ml fucoidan. Independent of the genetic background, addition of fucoidan further decreased cell viability over lapatinib alone (Figure 1C). Fucoidan doubled the killing activity of lapatinib, bringing the percentage of cell death form 30-40% by lapatinib, to 70-80% for the combination (Figure 1D), after three days of treatment. At 24 hours we also observed doubling of cell death, although to a lower degree, likely given the shorter treatment time, measured as the percent of sub-G1 population by cell cycle analysis (Supplementary Figure 1). Importantly, although the viability of normal human fibroblasts (BJs) was decreased (Figure 1C) indicating either decreased mitochondrial output and/or decreased growth, the drugs did not induce cell death (Figure 1D), even after exposure to the drugs for up to six days (not shown). These data would indicate tumor specificity of the treatment with negligible toxicity to normal cells.

### Fucoidan further decreases AKT and NFκB activity driven by lapatinib

The ability of fucoidan to interfere with a wide range of biological functions can in part be ascribed to its ability to interfere with several signaling pathways. For example, it has been shown to inhibit AKT [13], while it can either inhibit or promote ERK phosphorylation depending on the cell types [27, 28]. Also, it can affect angiogenesis by decreasing VEGF signaling [29]; and can decrease EGF induced EGFR phosphorylation [30].

**Figure 2:**
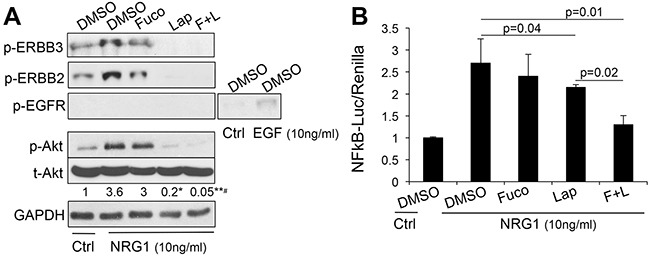
The combination lapatinib-fucoidan enhances the inhibition of AKT and NFκB Phospho-ERBB3, phospho-ERBB2, phospho-EGFR, total and phospho-AKT **A**. and NFκB activity **B**. measured in WM266-4 pretreated O/N with lapatinib (10μM), fucoidan (1mg/ml) alone or combined and then stimulated with 10 ng/ml neuregulin1 (NRG1) for 10 minutes prior protein lysate collection. EGF (10ng/ml) was used as positive control for phospho-EGFR. Data are representative of four independent experiments. Densitometric analysis of the band intensity was done using ImageJ. Fold change of band intensity is the ratio between phospho-AK T and GAPDH. Significant decrease in phospho-AKT upon lapatinib and lapatinib/fucoidan treatment was observed with respect to NRG1 stimulated cells: *p_LAP vs DMSO-NRG1_=0.0004; **p_L+F vs DMSO-NRG1_=0.0003; ^#^p_L+F vs Lap_<0.005, Student’s*t* test.

**Figure 3:**
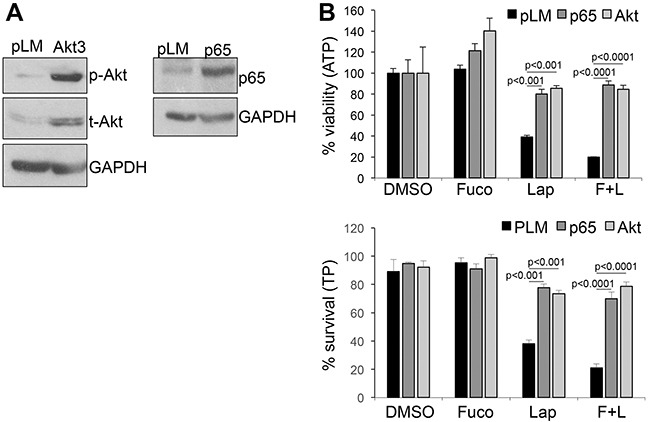
Over-expression of activated AKT and p65-NFκB rescues cell viability and survival **A**. expression of constitutive active Akt (left) and p65-NFκB (right) in WM266-4 cells. pLM: empty vector control. **B**. Viability (Cell titer glo-ATP) and survival (Trypan blue –TP) of the cells in A after three days in the presence of lapatinib (10μM) and fucoidan (1mg/ml) either alone or in combination. Significant differences in viability and survival, calculated by Student's *t* test, was observed in both AKT and NFκB over-expressing cells and both in lapatinib and lapatinib/fucoidan treatments.

Akt and NFκB are major survival pathways that we have shown play a major role downstream of ERBB signaling in melanoma [3]. Hence, we wanted to determine whether treatment with fucoidan in combination with lapatinib would further enhance the inhibitory activity of the latter on AKT and NFκB. As previously shown, lapatinib inhibited both factors (Figure 2A, 2B), however, the addition of fucoidan further decreased the activity of AKT and NFκB after stimulation with the ERBB3 ligand neuregulin1 (NRG1). Similar results were obtained in another melanoma cell line (Supplementary Figure 2). This suggests fucoidan enhances the killing capacity of lapatinib in part by increasing the suppression of these survival cascades downstream of ERBB3. Indeed, expression of a constitutively active AKT or of the NFκB isoform p65 (Figure 3A), rescued almost to the levels of controls, viability and survival of cells treated with lapatinb alone or with the lapatinib/fucoidan combination (Figure 3B).

The inhibitory effects on AKT signaling may be ascribed to the fact that fucoidan further inhibits the activation of ERBB3 and ERBB2 (shown as reduced phosphorylation in Figure 2A). On the other hand, we did not observe any signal from EGFR, another target of lapatinib, in cells stimulated with NRG1. EGFR was however activated by its specific ligand EGF (Figure 2A). This observation is intriguing and supports our previous finding of a key role of an ERBB3/ERBB2 signaling unit in triggering AKT signaling and survival in melanoma cells [2]. Indeed, when cells were subjected to EGFR specific (gefitinib) or ERBB2 specific (TAK165) inhibitors in combination with fucoidan, a synergistic inhibition of cell viability was only observed in cells treated with the ERBB2 inhibitor (Supplementary Figure 3). These data suggest fucoidan amplifies the effects of drugs that specifically inhibit the ERBB3/ERBB2 signaling pathway in melanoma.

### Addition of fucoidan enhances the antitumor activity of lapatinib and improves animal wellbeing

**Figure 4:**
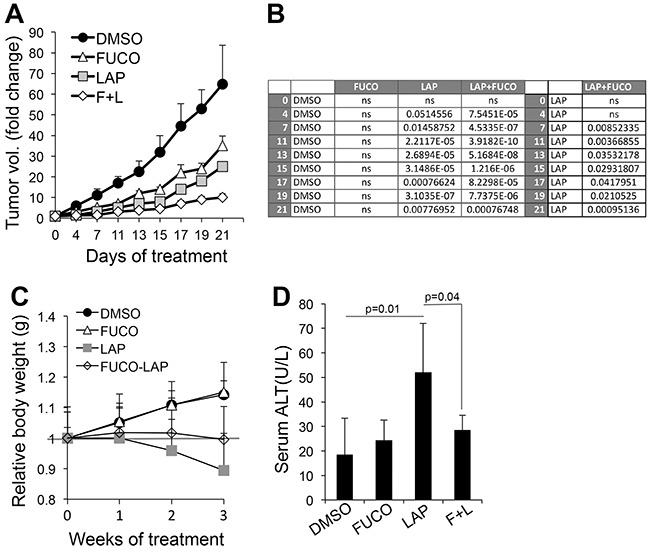
Fucoidan enhances the anti-melanoma effects of lapatinib **A**. Melanoma tumor growth of WM266-4 cells. Treatments were started when tumors in all animals measured an average 100-120 mm^3^ and were given 5 days/week for three weeks. **B**. Statistical analysis (Student's *t* test) at each time point comparing tumor growth of control (DMSO) vs Fuco, Lap and Lap/Fuco (left); and comparing Lap vs Lap/Fuco (right) **C**. Animal weight distribution over three weeks. The grey line refers to the relative weight at time 0. **D**. Alanine Transaminase (Liver toxicity marker) in serum (n=7 - DMSO and Fuco; n=15 - Lap and L+F). Significance between Lap and Lap/Fuco was calculated by Student's *t* test.

Given the intriguing *in vitro* results, we wanted to test whether the combination of fucoidan with lapatinib would enhance the anti-tumor effects of lapatinib *in vivo*. Immunodeficient mice were inoculated intradermally with WM266-4 cells. When tumors in all mice reached an average size of 100-120mm^3^, the animals were divided into four groups of treatments: 1) DMSO (Ctrl); 2) fucoidan (400mg/Kg s.c. [31]); Lapatinib (50mg/Kg I.P. [32]); 4) fucoidan+lapatinib. Mice were treated five days a week for three weeks and weighed once a week to assess their general wellbeing. The data show that fucoidan affected tumor growth, though not in a significant manner, whereas lapatinib inhibited growth roughly by 60% with respect to controls. Addition of fucoidan further decreased tumor growth to 85% (Figure 4A, 4B). Importantly, while a 10% body weight loss was observed in lapatinib treated mice (Figure 4C), the addition of fucoidan ameliorated the overall animal welfare as indicated by the maintenance of weight and diminished liver toxicity due to lapatinib (Figure 4C, 4D). These results suggest a therapy combining the ERBB inhibitor lapatinib at clinical therapeutic doses with the natural compound fucoidan can enhance the therapeutic benefits of Lapatinib while decreasing its side effects.

### ERBB3 inhibition recapitulates the effects of lapatinib

**Figure 5:**
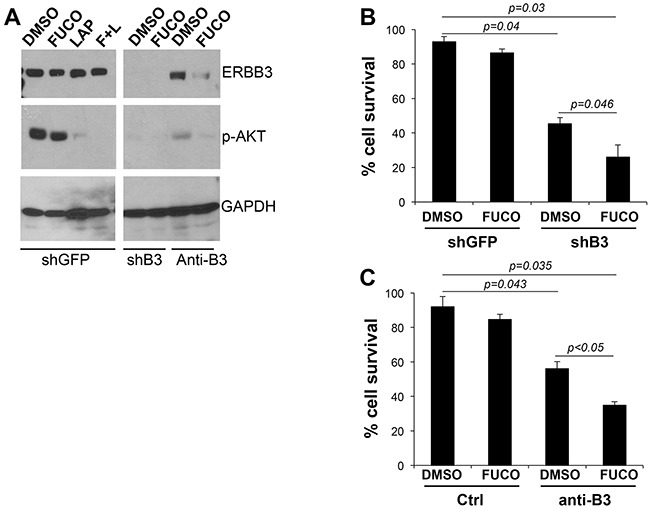
Fucoidan cooperates with specific inhibition of ERBB3 **A**. Expression levels of ERBB3, phosphor-AKT in cells expressing a specific shERBB3 (left panel) and cells treated O/N with a specific ERBB3 neutralizing antibody (10 ug/ml) (right panel). **B-C**. % survival (evaluated by trypan blue count) of cells (WM266-4) expressing shERBB3 and treated for three days with fucoidan (1 mg/ml) (B) or treated with anti ERBB3 neutralizing antibody (10 ug/ml) and fucoidan (1 mg/ml) alone or in combination (C). P values were calculated by Student's *t* test.

We have shown that lapatinib effectively inhibits the activation of ERBB3 [3]. We therefore wanted to determine whether specific ERBB3 inhibition could recapitulate the effects observed with lapatinb particularly when combined with fucoidan. We inhibited ERBB3 by either a specific shRNA (shB3) [2] or by using a novel ERBB3 neutralizing antibody (anti-B3) [24–26]. Both inhibitors were able to reduce AKT activation similarly to what we observed with lapatinib (Figure 5A). Inhibition of ERBB3 by a specific shRNA or by the anti-ERBB3 antibody reduced cell survival by approximately 30%, whereas fucoidan had negligible effects as previously observed (Figure 5B, 5C). However, addition of fucoidan further inhibited cell survival by 76% and 70% of sh-B3 and anti-B3, respectively.

Interestingly, inhibitors of other classes of kinases relevant to melanoma such as vemurafenib, which target mutated BRAF, did not synergize with fucoidan (Supplementary Figure 4), suggesting this natural compound amplifies the effects of inhibitors that target ERBB3 signaling.

## DISCUSSION

ERBB3 has recently emerged as an important player in melanoma. ERBB3 triggers survival cues to melanoma cells regardless of their oncogenic drivers [2]; ERBB3 is also a mechanism of resistance to BRAF inhibitors [8]; and promotes melanoma metastasis [9]. Hence, ERBB3 represents an intriguing target that can potentially affect multiple aspects of melanoma pathogenesis. Furthermore, the clinical availability of several inhibitors against ERBB signaling would support a fast translation to melanoma.

All members of the EBBB family of receptors are structurally very similar but have varied functional activity. EGFR and ERBB4, for example, have known ligands and active tyrosine kinase domains. ERBB2 and ERBB3 structurally and functionally cooperate with each other as ERBB2 has an active tyrosine kinase domain, but no known ligand, whereas ERBB3 has a specific ligand (NRG1) but lacks tyrosine kinase activity. Thus, ERBB2 remains in an open active conformation readily available for dimerization. In cells where PI3K is the major downstream signaling pathway, ERBB3 is the preferred dimerization partner for ERBB2 [7].

Interestingly, we have recently shown that PI3K/AKT and NFκB are major survival pathways downstream ERBB3/2 in melanoma [2]; and that lapatinib effectively inhibits ERBB2 and ERBB3 activation [3]. In this study we further show that ERBB3 is as major target of the lapatinib and fucoidan combination. Fucoidan, extracted from the New Zealand seaweed *U. pinnatifida*, doubles the cell killing capacity of lapatinib and increases tumor inhibition over lapatinib alone while significantly improving the side effects associated with prolonged lapatinib treatment. Although fucoidan alone did not significantly affect tumor growth, it did show a trend at tumor growth inhibition. It is possible that at higher doses, fucoidan would be more effective alone and possibly in combination with lapatinb. Future studies will determine the best effective dose of fucoidan *in vivo*.

Mechanistically, addition of fucoidan to lapatinib results in further reduction of AKT and NFκB activation. Reconstitution of AKT and NFκB function restores almost completely cell viability and survival, indicating these are key factors downstream of lapatinib/fucoidan. Additionally, we find that while ERBB3 and ERBB2 are affected by the treatment, EGFR was not, further reiterating our previous results that an ERBB3/ERBB2 signaling cascade operates in melanoma and is mostly responsible of the activation of PI3K/AKT signaling [2]. Indeed, specific inhibition of ERBB2 recapitulated the effects observed with lapatinib, whereas fucoidan in combination with gefitinib, a specific EGFR inhibitor, did not exert any synergistic effects on cell viability.

That ERBB3 is a major target of the combination treatment is further confirmed by recapitulation of some of these effects when ERBB3 is specifically knock down or blocked using specific shRNAs against ERBB3 or a selective ERBB3 neutralizing antibody. Overall these data indicate a combination involving the natural compound fucoidan and ERBB3 inhibition is a promising novel and safe approach in melanoma therapy.

Lapatinib has been approved for the treatment of breast cancer patients and a phase II clinical trial of lapatinib for the treatment of stage IV melanoma harboring ERBB4 mutations has recently been concluded (NCT01264081). Although analysis of the results was possible for a small fraction of the recruited patients, it did show stable disease in those patients. Nevertheless, it also demonstrated that lapatinib works better when in combination with other therapies in several cancers. For example, Lapatinib, in combination with capecitabine, has shown to extend time to disease progression compared to chemotherapy alone in advanced HER2(+) breast cancer patients that have progressed on trastuzumab [33]. In melanoma, it has been shown in preclinical models that the combination of lapatinib with the BRAF inhibitor PLX4720 reduces tumor burden and extends latency of tumor regrowth *in vivo* versus PLX4720 alone [33]. We have recently reported that combination of lapatinib with a γ-secretase inhibitor (GSI), to concurrently inhibit the ERBB and Notch pathways, promotes melanoma regression, whereas the use of either inhibitor alone can only delay tumor growth [3]. Hence, combination therapies have become standard of care for most cancer patients including melanoma patients [34].

Yet, while effective, the interaction of different agents in combination therapies may add further toxicities and may require a more careful management. Lapatinib for example, although generally considered well tolerated, can lead to gastrointestinal side effects such as diarrhea, nausea and vomiting that can result in weight loss, skin rash and fatigue, which, in the long-run can affect the quality of life of patients [10] [35]. Hence, means to potentiate lapatinib efficacy while improving its tolerability would be highly desirable in the clinic.

Fucoidan has been shown to synergize with standard anti-cancer agents and/or reduce their side effects [23]. For example, fucoidan enhances the efficacy of cisplatin, tamoxifen and paclitaxel [36] while improving their toxicities [37]. In our study, we observed a strong synergy between lapatinib and fucoidan such that addition of fucoidan increased the anti-tumor properties of lapatinib and importantly, improved the animal well-being. Mice did not lose any weight throughout treatment and the levels of ALT, a marker of liver toxicity that is increased in a subset of patients treated with lapatinib [38], decreased. Therefore, adding fucoidan to an ERBB based therapy may prove extremely useful particularly in those patients whose liver toxicity can be life threatening to the point of requiring suspension of the treatment.

Finally, it is worth noticing that *U. pinnatifida* is an unwanted organism that has infested and spread in several areas in New Zealand such that the harvest and use of it is encouraged and provides a relatively cheap source of fucoidan to be used in combination with anti ERBB therapies in melanoma and possibly other cancers.

## MATERIALS AND METHODS

### Cells lines

Melanoma cells used in this study were: WM266-4, WM115 (mutated BRAF), SKMEL2 (RAS mutated), MeWo and FEMX (wild type). BJ fibroblasts were obtained from ATCC (ATCC-CRL-2522). All cells were maintained in DMEM (Dulbecco's modied Eagle's medium) supplemented with 10% fetal calf serum, 1% glutamine and 1% penicillin–streptomycin.

### Crude fucoidan extraction

Sporophyll of seaweed *Undaria pinnatifida* samples were harvested from Marlborough Sounds, New Zealand. The samples were then processed as in to extract crude fucoidan. Briefly, sporophylls were dried and treated at room temperature for 24 h with a MeOH–CHCl_3_–water mixture (4:2:1) to remove lipids, protein, and colored pigments. The treated algal biomass was filtered through a Whatman's filter paper (90 mm GF/D), then washed with acetone and dried overnight at room temperature. Treated material was mechanically stirred with 2% aqueous CaCl_2_ (100 mL) at 85°C for 5 h. The extract was centrifuged at 18500 *g* and the supernatant was collected. Pure ethanol was added to the extract to make the final ethanol concentration to reach 70% and the mixture was left to precipitate at 4°C overnight. The precipitate was centrifuged at 18500 *g*, washed with water and mechanically stirred with 60 mL ethanolic NaI solution (20%) for 72 h. The precipitate was removed by centrifugation, washed with ethanol and lyophilized to give crude fucoidan. An acqueous solution of fucoidan was used in both invitro and *in vivo* experiments.

### Plasmids, shRNAs and chemicals

shRNAs against human ERBB3 (TRCN0000000621) was purchased from sigma (Saint Louis, MO, USA). Lapatinib was from LC Laboratories (Woburn, MA, USA). All compounds were dissolved in DMSO (Sigma, Saint Louis, MO, USA).

### Luciferase assays

WM266-4 cells (5 × 10^4^) were transfected with a NFκB reporter construct [39] and a CMV-driven Renilla reporter control at a 1:20 ratio, respectively. After 36–48 h, activities of Firefly and Renilla were assessed by the dual-luciferase Assay system (Promega, Madison, WI, USA)) and light production was measured for 10 s in a Monolight 2010 Luminometer (Molecular Devices, Sunnyvale, CA, USA).

### Western blot analysis

Serum starved cells (2 × 10^6^) were treated with vehicle (DMSO) or compounds at the indicated doses and with 10ng/ml neuregulin 1 (NRG1), and were then collected 24 h post-treatment. Total protein was extracted with urea lysis buffer (9 M urea; 75 μM Tris–HCl, pH 7.5 and 100 μM 2-mercaptoethanol (2-ME)). 40–50 μg per sample were separated by 8–10% sodium dodecyl sulfate polyacrylamide gel electrophoresis and transferred onto nitrocellulose membranes. Antibodies against phospho-EGFR, phospho-ERBB2, total ERBB3 and total and phosphor-AKT were from Cell Signaling Technologies (Beverly, MA, USA). Loading was normalized with anti GAPDH (Santa Cruz Biotechnology).

### Viability and cell death assay

Viability was evaluated by the Cell titer-Glo (Promega, Madison, WI, USA) as per manufacturer's instructions on 3000 cells seeded in triplicate in 96-well plates and treated for three days with several doses of fucoidan and 10 μM lapatinib. Cell death was calculated on cells treated as above by using trypan blue.

### *In vivo* tumor growth

Cells (2 × 10^6^) were injected subcutaneously in the dorsal flanks of 8 weeks old Male SCID mice for a total of ten tumors and fifteen tumors per DMSO, Fucoidn and Lapatinib, Lap+ Fuco, respectively. Mice were supplied by the Athymic Animal Facility at Case Western Reserve University under an Administrative Panel on Laboratory Animal Care approved protocol. Tumors were measured with a caliper and tumor volumes calculated. Treatments were started when tumors in all animals reach an average size of 100-120 mm^3^. Mice were divided into four groups of treatments and received the following doses of compounds five consecutive days a week: 1) DMSO; 2) fucoidan (400mg/Kg s.c. [31]); Lapatinib (50mg/Kg I.P. [32]); 4) fucoidan+lapatinib.

### Alanine transaminase assay

100 ul blood was collected from the tail vein one our after the last treatment. Blood was centrifuged at 1000 × g and serum collected. 20 ul of serum from each animal was used for the analysis using the Enzychrom™ Alanine Transaminase Assay Kit, as per manufacturer's instructions (Bioassay system, Hayward, CA).

## SUPPLEMENTARY MATERIALS FIGURES


