# Supplementary material for: The natural compound fucoidan from New Zealand Undaria pinnatifida synergizes with the ERBB inhibitor lapatinib enhancing melanoma growth inhibition
Source: Oncotarget. 2017 Jan 2;8(11):17887–96. doi: 10.18632/oncotarget.14437 (PMC5392294; doi:10.18632/oncotarget.14437)
Supplement: Supplementary file 1 [file oncotarget-08-17887-s001.pdf]

The natural compound fucoidan from New Zealand *Undaria pinnatifida* synergizes with the ERBB inhibitor lapatinib enhancing melanoma growth inhibition

SUPPLEMENTARY FIGURES

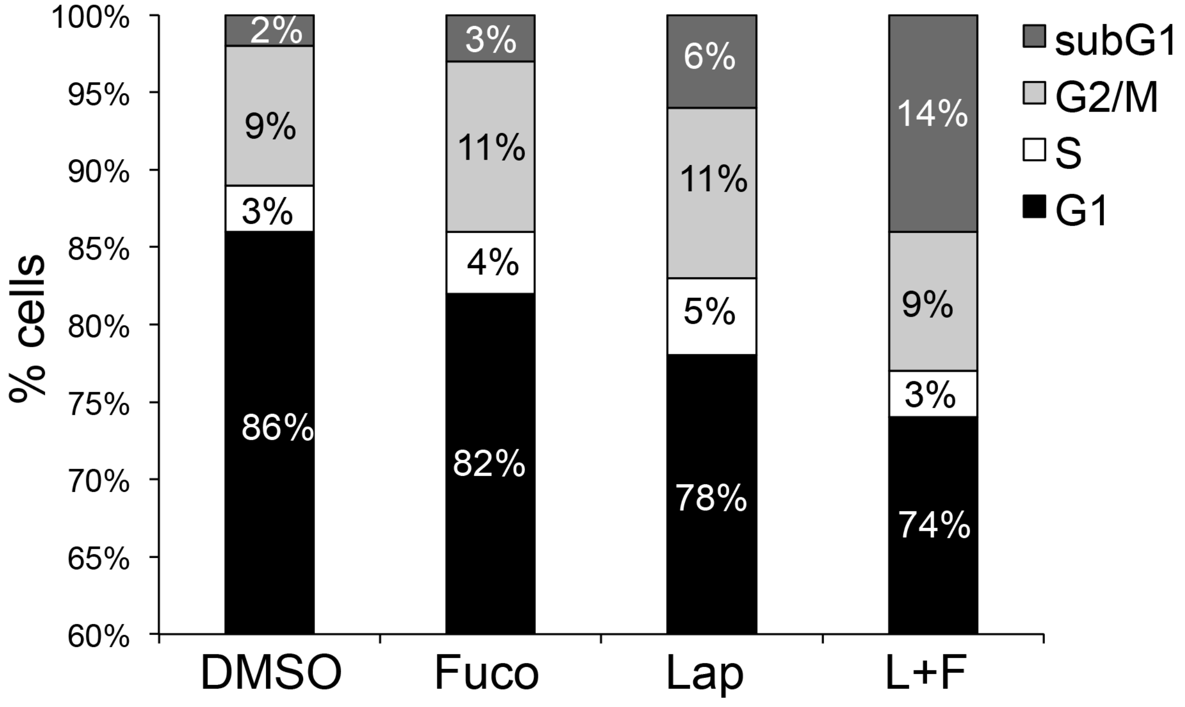

Supplementary Figure 1: cell cycle analysis of WM266-4 cells treated with lapatinib and fucoidan. Cells were seeded for 24 hours prior to 24 hours treatment with 10µM lapatinib and 1mg/ml fucoidan, alone or combined. Cells were collected, fixed with 90% methanol and washed twice in ice-cold 1% bovine-serum albumin in PBS. RNA was removed with 2.5 mg/ml Dnase-free RNAse-A, and DNA was stained with 20 mg/ml propidium iodide (PI). The fluorescence of PI stained was measured with an EPIC-XL flow cytometer (Coultier, Miami, FL).

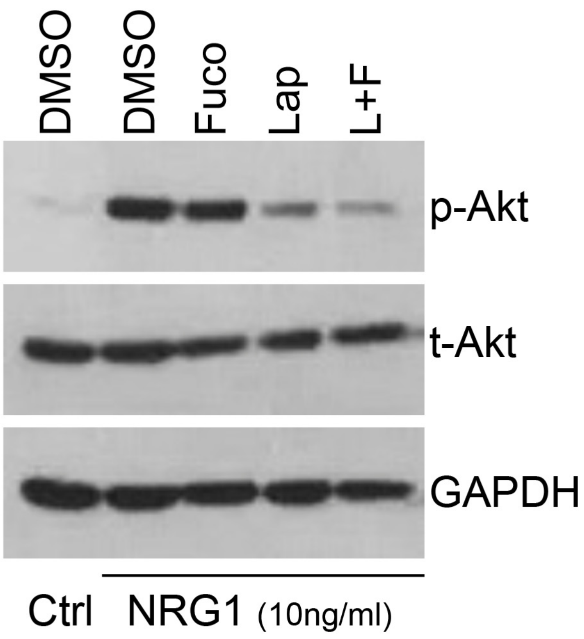

Supplementary Figure S2: Akt phosphorylation upon lapatinib and fucoidan treatment in K457 melanoma cells. Cells (10^6) were seeded in complete media then serum starved O/N in the presence of 10µM lapatinib and 1mg/ml fucoidan, alone or combined.

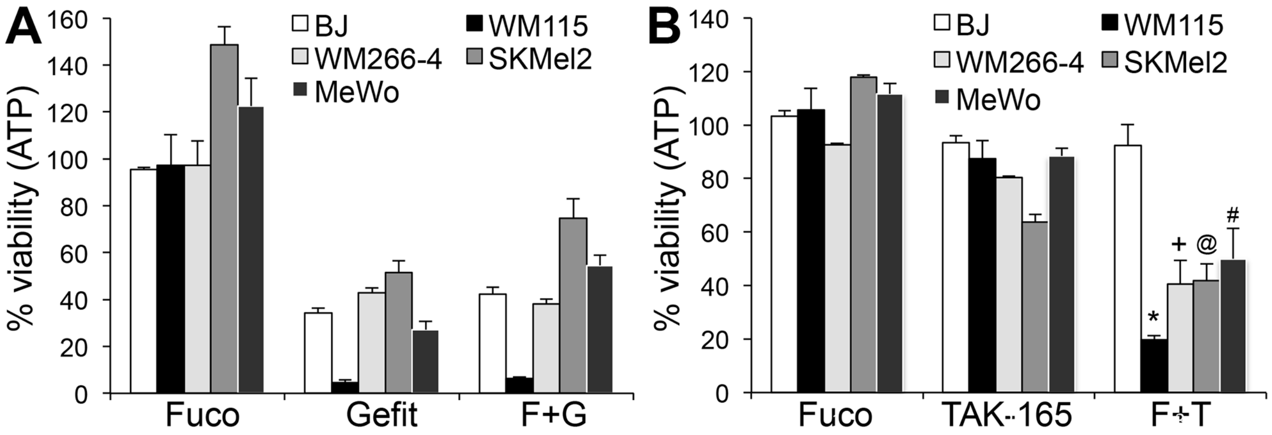

Supplementary Figure 3: Only inhibition of ERBB2 synergizes with fucoidan. Viability of BRAF mutated (WM115, WM266-4), Ras mutated (SKMel2) and WT/WT (MeWo) melanoma cells and of human fibroblasts (BJ), treated for three days with gefitinib (20µM) (EGFR inhibitor) and fucoidan (1mg/ml) **A.** or with TAK-165 (50nM)(ERBB2 inhibitor) and fucoidan (1mg/ml) **B.** alone or combined. *p<0.0001; +p<0.01; @p=0.02; #p<0.05.

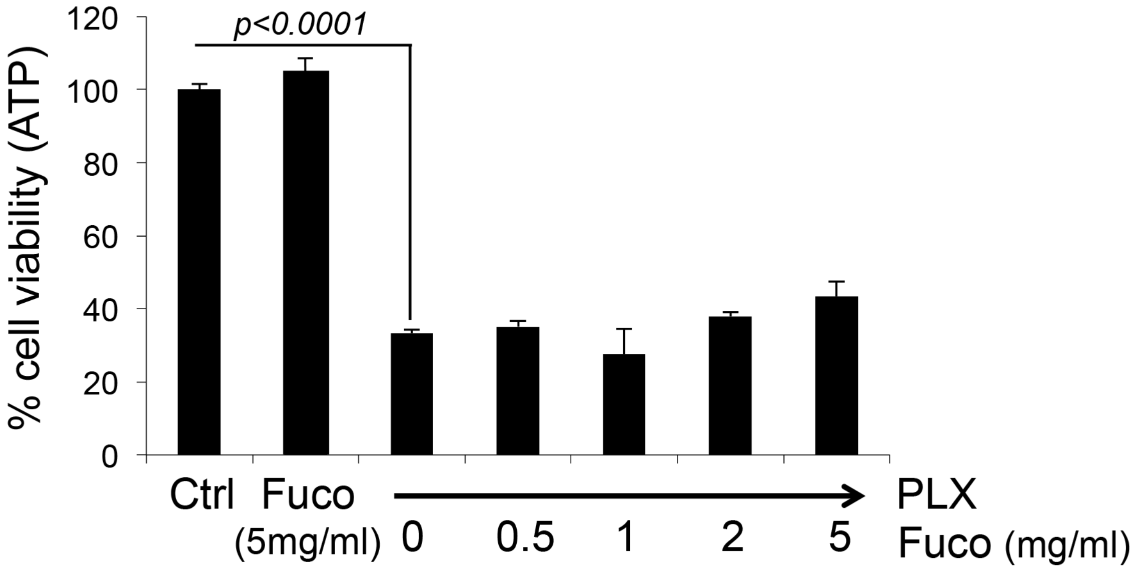

Supplementary Figure 4: Fucoidan does not enhance the inhibitory effects of the BRAF^{mut} inhibitor Vemurafenib (PLX4720). Cells (WM115) were treated for three days in the presence of the indicated compound prior to measurement of viability by the Cell titer glo assay. Fucoidan did not enhance the effects of PLX4720 at any dose used.
